# Ethical psychiatry in an uncertain world: conversations and parallel truths

**DOI:** 10.1186/1747-5341-4-7

**Published:** 2009-06-25

**Authors:** Alexander M Carson, Peter Lepping

**Affiliations:** 1 School of Health, Social Care, Sport and Exercise Sciences, Glyndwr University, Plas Coch, Wrexham, UK; 2 North Wales NHS Trust & Cardiff University, Wrexham Academic Unit, Technology Park, Croesnewydd Road, Wrexham LL13 7YP, UK

## Abstract

Psychiatric practice is often faced with complex situations that seem to pose serious moral dilemmas for practitioners. Methods for solving these dilemmas have included the development of more objective rules to guide the practitioner such as utilitarianism and deontology. A more modern variant on this objective model has been 'Principlism' where 4 mid level rules are used to help solve these complex problems. In opposition to this, there has recently been a focus on more subjective criteria for resolving complex moral dilemmas. In particular, virtue ethics has been posited as a more sensitive method for helping doctors to reason their way through difficult ethical issues. Here the focus is on the character traits of the practitioner. Bloch and Green advocated another way whereby more objective methods such as Principlism and virtue ethics are combined to produce what they considered sound moral reasoning in psychiatrists. This paper points out some difficulties with this approach and instead suggests that a better model of ethical judgment could be developed through the use of narratives or stories. This idea puts equal prima facie value on the patient's and the psychiatrist's version of the dilemma they are faced with. It has the potential to lead to a more genuine empathy and reflective decision-making.

## Introduction

In professions that have direct contact with people, the role of humanities in professional education assumes a particularly important value. Since the vast majority of the work of health care professionals is with colleagues and clients, it seems obvious that humanities in general and ethics in particular should play a large part in both their education and their clinical practice. Doctors have traditionally viewed the Hippocratic Oath as an ethical framework in which to practice medicine but as medicine has become more complex, so has its ethical dilemmas. There has been a great deal of discussion about whether medicine in general and psychiatry in particular are faced with such unique circumstances in clinical practice that they need a unique ethical framework [1]. In a recent, thoughtful article, Sidney Bloch and Stephen Green not only agree that psychiatry needs an ethical framework that can capture the complex moral dilemmas inherent in practice but they also provide a framework that provides a complementary model of ethical practice [2]. Their model is designed to link Principle based ethics with virtue ethics. This mix or complement of objective rules or Principles and subjective character traits is, they contend, a method of practitioners exercising what could be called, 'judgment within limits'. Principles, according to Bloch and Green, provide the boundaries or limits in which practitioners can exercise their judgments. To give justice to the actual situation or relationship they advocate the use of character based ethics to provide the emotional core or 'heart' to the ethical decision. Their model is a combination of mind and heart that tries to complement each other in attempting to resolve difficult moral dilemmas. While recognizing problems with both Principlism and virtue ethics per se, Bloch and Green assert that they can, in combination, provide a sound moral framework 'based on conceptual compatibility and synergy'. In the next section of this paper, we point out some problems, both methodological and practical, with this position and suggest a way forward.

## Ethics: The heart of the problem

Bloch and Green make an important point when they say that we need to put 'heart' into ethical decision making. They think that a principle-based approach to ethical decision making leaves out too much of the personal in delicate situations. They propose an additional or 'complementary' framework which, they say, can make us more sensitive to the real situation. To illustrate the complexity of clinical situations, they use a clinical scenario which they call 'Jill, Tim and the baby'. They then explore various possible ethical solutions to the scenario and find all the usual methods problematic. Deontological approaches, they assert, cannot resolve moral conflicts and so the psychiatrist is 'denied an available remedy'. Utilitarianism is seen by the authors as too difficult to calculate benefits and risks and demands an impartiality that clinicians would find difficult to achieve. Both deontology, a respect for patient autonomy, and utility, a measurement of consequences, are seen as theories that do not help clinicians in practice. This is particularly the case in conflict situations. For example, an older person may want to stay in her home despite the risk she might pose to herself and others. Deontology would argue that we should respect the patient's views while utilitarianism argues that we should decide the case, based on possible consequences [3]. Neither theory can resolve a complex clinical situation such as this as both are in conflict.

The authors then go on to examine the value of Principle-based ethics as a middle way approach to ethical dilemmas. Their problem with this approach is that while it does provide ethical guidelines, the approach is 'far from definitive'. Moral reasoning, they contend, based on a principled approach, 'falls between the poles of subjectivity and objectivity'. They then turn to virtue ethics as a possible way of producing good ethical decisions in clinical practice. Virtue ethics, derived from Aristotle, links persons and actions in a virtuous circle. The idea is that a cultivation of ethical qualities or character traits will lead clinicians to act ethically in clinical situations. One difficulty which they identify with this approach is that there is no clear understanding of how these ethical or virtuous characteristics are developed in people, whether they are genetically or socially derived. They conclude that virtue ethics, by itself, 'cannot.....guide clinicians to deal with the moral complexity facing them'. In their search for a possible way of resolving difficult ethical cases, they finally turn to a variation on virtue ethics, the ethics of care. Here, they assert, emotions have a part to play in moral reasoning. However, they find that too much reliance on emotions will just produce subjective judgments that undercut any attempt to produce 'reasoned ethical debate' and will instead produce a relativism, in which everyone is equally right. In summary they find that all single approaches to the development of sound ethical reasoning in complex clinical situations are problematic. They propose instead, a potential remedy to this problem.

They see the work of Annette Baier as part of a possible way of developing sound moral reasoning in clinical situations. Baier, they suggest, sees contributing to 'a climate of trust' as a primary responsibility for clinicians, particularly psychiatrists, in clinical situations. Promoting trust between clients and clinicians, they argue, is at the heart of all clinical situations. However, they also argue that this should be complemented by 'a more structured framework', namely, Principlism. This mix of guiding principles and a context of trust, they argue, will provide clinicians with the opportunity to examine 'the ethical nuts and bolts' of clinical situations through sound moral reasoning. While this provides a more sensitive approach to complex clinical encounters, it has its own difficulties

## The scenario and the narrative

If we go back to Bloch and Green's scenario, we can perhaps see the problem. A consultant psychiatrist, Dr Jones, has to choose between enforced treatment for a woman who does not see the need for her to have any medical care. The authors explore this scenario with their new complementary model. They show that a climate of trust must first be built up, using a character-based approach, to extend care to the family. This can enhance Dr Jones' empathy and understanding of the family but it is not enough to approach the 'level of clarity required to reach reasoned moral judgments'. Here the four principles can be used to structure the moral deliberations that Dr Jones will make. Bloch and Green explore the scenario using these principles and conclude that Dr Jones may have to act paternalistically and treat the woman. Although, they acknowledge that whatever intervention is finally decided on by Dr Jones, there will be a degree of uncertainty in outcome, they assert that they have provided a 'means to reflect iteratively on what constitutes the most apt ethical action'. We would agree, with Bloch and Green that outcomes are not necessarily everything, but we do have problems with their approach.

The first difficulty is that while Bloch and Green criticize others for developing ethical *methods *rather than *theories*, they do the same thing. The paper is called 'An ethical framework for psychiatry' but it is, in practice, a method or procedure. It lays out a series of procedural steps for exploring ethical dilemmas but it does not ask itself about the 'ethical dilemma' itself. This is because the iterative process is carried out by the doctor without really hearing from the woman. Part of the reason for this is that the doctor implicitly relegates her views or story, as she is 'disturbed'. The scenario is already paternalistic before any further exploration is carried out; this doctor already 'understands'. The procedure he then adopts only confirms him in his paternalism. If it was really a critically reflective process, the doctor could see that. Our problem with Bloch and Green's framework is that it is not reflective enough and could simply confirm a psychiatrist's original impressions and outlook. We do not hear from Jill or Tim, only from the doctor.

Barons's work demonstrates that doctors may be distracted from seriously listening to clients by their need to follow the structured 'listening' inherent in the medical model itself [4]. This kind of listening is a kind of detective model in which client's narratives are re-structured within assumptions and theories that professionals bring to the clinical encounter. As previously mentioned, a recent paper makes the point that psychiatric nurses fit what they hear from clients into their therapeutic contexts [5]. A family doctor, for example, may 'hear' a client's complaint about a headache as a 'neurological disturbance'. Both participants in the conversation may, in practice, be talking about different things. Clients' narratives might be automatically fitted into diagnostic criteria, as Bloch and Green do in their example, where the client is already 'delusional'. So although most doctors would claim that they really do listen to their clients, we suspect that this listening is already pre-judged, Bloch and Green's ethical framework could be seen as another professional model of judgment that simply fits clients' points of view into something prepared earlier. Our version of active listening is an appeal for more genuine openness and empathy from doctors.

While Bloch and Green do make a sincere attempt to empathize with Jill and Tim, it is a little difficult to validate this since we do not know what their empathy would look like. It might be convincing the couple that the doctor really does have the couple's best interests at heart but Jill and Tim cannot even say this. This is the heart of the matter. Bloch and Green want the patient or client to trust them that they have their best interests at heart as they have a procedure for thinking about these complex ethical situations. What might help us to trust them is if the doctor allowed us to hear from Jill and Tim. They, perhaps, would see things differently. Bloch and Green ask the same of their readers as they do of their clients: trust me, I'm a doctor. However, they don't really give us or their clients a reason for doing so. This is a problem with case studies and clinical scenarios that has already been discussed by the first author [6]. If we are going to explore complex ethical situations, we have to see the people involved and hear their story. The clinical scenario tells us about the doctor and his story. It does not allow the doctor to reflect on his story and the other possible stories that Jill and Tim may have told. They may have contradicted or confirmed the story, but we shall never know; we are required to trust the doctor. While Bloch and Green claim that their model provides clinicians with a 'means to reflect iteratively on what constitutes the most apt ethical action', there is no real iterative process, just the doctor talking to himself.

## Other persons, other stories

What seems to be missing from Bloch and Green's model is any real sense that there may be other stories to tell and other stories to hear. In their model, the doctor sees both sides and makes ethical decisions weighing up 'both sides'. As has already been pointed out, this is not an authentic 'other' side, simply the doctor imagining it. However, in all professional/client encounters there are always two sides to everything. Given this, we need to think that a doctor could be wrong in a more fundamental way than Bloch and Green imagine. It is not simply a matter of doctors making the wrong ethical decision; this is always a possibility. Bloch and Green try to provide a procedure for minimizing these mistakes. But we are equally not arguing merely for standardized best practice. Whilst standardized best practice is laudable in itself, our criticism is not a criticism of poor practice. It is a much more fundamental criticism of the way ethical decisions are being made in a prescribed process-driven manner.

The deeper problem for Bloch and Green's ethical model is that the doctor could be seeing the situation wrongly. In their scenario, the doctor tests *his *view of the situation against *his *view of how the woman sees it. What seems to get in the way is the doctor's sense that his client(s) is mentally disturbed and this will, we believe, lead to an 'instinct to mistrust' the client. Whilst skepticism in the validity of the patient's story may be justified in many cases, in will not be justified in some. However, it will not be true in every case. In a recent book, one of the authors pointed out this problem for mental health nurses who base their practices on the assumption that all their clients *need *therapy [5]. In this kind of 'procedural' practice, the focus is on what kind of therapy rather than on the initial issue of whether clients need therapy in the first place. Bloch and Green are caught up in this procedural debate assuming that their view of the situation is the only way to see things. In this model there is no empathic relationship between doctor and client.

If psychiatrists are going to practice ethically, they have to begin with the assumption that their clients have an equally valid point of view to the doctor's and have to be prepared to be wrong in their view of the situation. This goes far beyond standardized current best practice. It is not enough for the psychiatrist to have a set of Principles and a virtuous character, though these are useful attributes. Ethical psychiatry is more about initially according equal weight to the possibility that the client might have a story to tell. Here Ross's notion of parallel truths can be helpful [7]. Ross wants to show that people operate with different parallel truths, which they use to interpret the world and situations they find themselves in. In practice, this means that psychiatrists and clients can potentially operate within different moral universes and 'forms of life' [8]. If we are going to develop a truly ethical psychiatry, it cannot be one where the psychiatrist does all the imagining and evaluating, in other words: being judge and jury in one person. Ross points us to the fact that people see things differently but from equally valid points of view. If we are to take this seriously we need to develop a clinical method that values this notion of 'parallel truths' as a beginning for any clinical encounter.

What is needed is a way of engaging both psychiatrists and clients in conversations where there is an assumption that both have equally valid stories to tell. This is in keeping with our assumption that there is no absolute truth and no absolutely right or wrong decision when it comes to ethical dilemmas. This conversational model gives equal initial weight to all narratives, both psychiatrist's and client's.

## Ethical psychiatry and ethical conversations

We have a problem with the notion of psychiatric ethics. Instead, we prefer the term 'ethical psychiatry'. Psychiatry is about helping people who suffer from mental illness. Here, the ethic of helping people comes first. This ethic is the foundation of psychiatric practice. Although this is a banal truism, it is often overlooked in everyday practice. This is because practicing psychiatrists often see their everyday practice as 'problem solving'. However, solving problems is not the same thing as helping people. In many ways, psychiatrists work much more closely with their clients than other clinicians. They are often faced with a complex mix of technical, social and personal problems. This is in contrast to more 'technical' clinical practices such as surgery and so on. After all, it is unlikely that clients have strong views about particular surgical techniques. Given this, it is even more important that psychiatrists develop a genuinely iterative process in working with clients. We suggest a particular conversational framework that might help psychiatry to practice in a more ethically reflective way.

Charles Taylor points out:

When we see something surprising, or something that disconcerts us, or which we can't quite see, we normally react by setting ourselves to look more closely: we alter our stance, perhaps rub our eyes, concentrate, and the like [9].

This is something that we all do from time to time. We sometimes see something that disrupts our normal perceptions. Here we question our first impressions. The process involves us moving around, checking our equipment, getting more focused and so on. In a similar way our conversational framework allows different perceptions or views of the situation to question each other. In clinical practice, the medical staff's view of the situation or problem could be questioned by the client's alternative story. Many doctors would want to argue that they do allow their clients' views to influence their clinical judgments. We would certainly agree with that. However, the point we are making is slightly different: The notion of parallel truths provides firmer ground for conversations between doctors and clients. It enables the doctors to take seriously the client's story rather than automatically devaluing it with their own story. We are not suggesting that psychiatrists and other doctors have not a good story to tell; we are simply arguing that the client may have an equally good one. If we assume this, we need to develop ways of seriously engaging with this. In many situations, the doctor's view of the situation will prevail; but not *always*. Our conversational model assumes an initial validity for each story. This allows the practitioner's view of the situation to be explicitly challenged by the client's. By listening seriously to the client's story, it allows the clinician to get closer to the client as a person. It allows the clinician to engage in 'disciplined empathy' with the client. Disciplined empathy is about *seriously *and reflectively listening to the client's story.

The client's story provides an opportunity and a context for genuine empathy between professional and client. Empathy, on Bloch and Green's model is about the doctor doing all of the work by imagining things from the client's point of view. Seriously listening to the client's story gives the doctor a real insight into the client's life context and thus frames and disciplines his or her response. It provides a context for the doctor's deliberations and ongoing conversations with the client. It also provides an opportunity for clinicians to reflect on their own practice by seeing it from the client's point of view and could help in the development of clinical skills and ethical competence. Just knowing ethical theories will not necessarily make a clinician a good ethical practitioner. As well as developing ethical competence, treating patients' stories as of equal validity, will allow practitioners to gain a stronger degree of empathy with their clients life and provide valuable insights into their own practice.

## Conclusion

In Bloch and Green's scenario, using narratives and parallel truths would have allowed Dr Jones to reflect on Jill and Tim's story. Valuing those stories as equal would have helped to create empathy that allowed better reconciliation between Jill's views and the available treatment options. It would have avoided a cognitive framework which forced Dr Jones into looking at Jill's point of view entirely through the eye of an illness model, as well intended as this may have been.

In this paper, we have tried to suggest a more ethically aware framework for psychiatric practice in particular and medicine in general. The framework is designed to be sensitive to each clinical encounter by enabling doctors to engage with their clients in serious conversations where each point of view or narrative is seen as equally valid. Underpinning this framework is the ethic of helping people. While not prescriptive, this ethic can help to evaluate these narratives. The ethic becomes part of the conversation as it provides a standard or measure in judging each narrative. Judgments in clinical practice are a matter of reconciling available treatment options with individual clients. This narrative based conversation can help doctors make more ethically conscious judgments by getting them to see their initial impressions from a different perspective. It provides a reflective opportunity for a clinician to examine his or her own practice

We are not suggesting that ethical psychiatry or medicine is about clinicians giving up responsibility for their judgments in practice. We would imagine that in the majority of cases, clinicians' experiences would be the deciding factor. However, our framework for the practice of a genuinely ethical psychiatry assumes that the clinician's experience may not always be the deciding factor. A clinician, using our framework, is open to this possibility. We do not provide any answers to particular situations but we suggest that a genuinely ethical psychiatry and medicine should begin with this assumption. By listening to a client's story, a clinician may gain some insight into the person behind the client role and that would really be about helping people. Narratives and story-telling are something that we can all engage in. They emphasize the key role that the humanities in particular can play in the education of psychiatrists, doctors and health care professionals.

## Competing interests

The authors declare that they have no competing interests.

## Authors' contributions

Both authors contributed equally to the text.

## About the authors

AMC is a qualified psychiatric nurse. He changed clinical practice for an academic career and is now Head of School at the School of Health, Social Care, Sport and Exercise Sciences at Glyndwr University in Wrexham, Wales, United Kingdom. He has a PhD in ethics and has widely published on narratives in ethical practice.

PL went to University in Münster, Germany. He has been working in the United Kingdom since 1995. He has a Masters in medical ethics and won several research prizes. His research interests include violence and aggression, delusional infestation, ethics, risk mitigation and various other projects. He has honorary academic appointments with the University of Wales, Bangor and Cardiff University. He works as a consultant psychiatrist in Wrexham, North Wales. He runs a specialist clinic for adult ADHD. He is also the Associate Medical Director for ethics, capacity and consent for the North Wales NHS Trust.
